# Positive association between metabolic syndrome and bone mineral density among Malaysians

**DOI:** 10.7150/ijms.49030

**Published:** 2020-09-16

**Authors:** Kok-Yong Chin, Chin Yi Chan, Shaanthana Subramaniam, Norliza Muhammad, Ahmad Fairus, Pei Yuen Ng, Nor Aini Jamil, Noorazah Abd Aziz, Soelaiman Ima-Nirwana, Norazlina Mohamed

**Affiliations:** 1 Department of Pharmacology, Universiti Kebangsaan Malaysia Medical Centre, Cheras 56000, Malaysia.; 2 Department of Anatomy, Universiti Kebangsaan Malaysia Medical Centre, Cheras 56000, Malaysia.; 3 Drug and Herbal Research Centre, Faculty of Pharmacy, Universiti Kebangsaan Malaysia Kuala Lumpur Campus, Jalan Raja Muda Abdul Aziz, Kuala Lumpur 50300, Malaysia.; 4 Centre for Community Health Studies (ReaCH), Faculty of Health Science, Universiti Kebangsaan Malaysia Kuala Lumpur Campus, Jalan Raja Muda Abdul Aziz, Kuala Lumpur 50300, Malaysia.; 5 Department of Family Medicine, Universiti Kebangsaan Malaysia Medical Centre, Cheras 56000, Malaysia.

**Keywords:** cholesterol, hypertension, insulin resistance, osteopenia, osteoporosis, triglycerides

## Abstract

**Objectives:** Metabolic syndrome (MetS) is a cluster of metabolic abnormalities that elevates the individual risk of cardiovascular diseases. These abnormalities are also known to alter bone remodelling. Therefore, MetS may be associated with osteoporosis. This study aims to determine the association between MetS and its components and bone mineral density (BMD) assessed by dual-energy X-ray absorptiometry (DXA) among Malaysians.

**Methods:** 400 Malaysians aged ≥ 40 years (52.5% women) residing in Klang Valley, Malaysia, were recruited. Subjects' demographic and lifestyle details were collected using a questionnaire, and blood pressure and body anthropometry were measured. Subjects' lumbar spine and total hip BMD were measured by DXA. Their fasting blood was collected for blood glucose level and lipid profile analysis. Regression analysis was used to analyze the relationship between MetS or its components and BMD.

**Results:** Subjects with MetS had higher BMD compared to subjects without MetS in models unadjusted for BMI (spine *p=*0.008; hip *p<*0.001). This difference was attenuated with BMI adjustment (spine *p=*0.625; hip *p=*0.478). Waist circumference was associated positively with BMD in models unadjusted for BMI (spine *p=*0.012; hip *p<*0.001), but the association became negative with BMI adjustment (spine *p=*0.044; hip *p=*0.021). Systolic blood pressure was associated positively with total hip BMD (*p=*0.019) but BMI adjustment attenuated the relationship (*p=*0.080). Triglyceride level was associated with osteoporosis in a fully adjusted model (*p=*0.001). Overall, MetS was associated with osteoporosis (*p=*0.019) but lifestyle (*p=*0.188) and BMI adjustment attenuated the relationship (*p=*0.904).

**Conclusion:** MetS is positively associated with BMD, and this relationship is predominantly mediated by BMI. Although MetS is not a significant risk factor for osteoporosis, the inverse relationship between waist circumference, a marker of central obesity, and BMD highlights the need to prevent adiposity to improve metabolic and skeletal health.

## Introduction

Metabolic syndrome (MetS) is a collection of medical conditions, such as central obesity, hyperglycaemia, hypertension and dyslipidaemia, which elevate the risk of diabetes mellitus and cardiovascular diseases when occurring together 1. Various organizations have put forward different definitions of MetS throughout history 2. The latest set of criteria comes in the Joint Interim Statement (JIS) by these organizations aiming to unify the definitions 3. Based on the JIS criteria, 42.5% [95% confidence interval (CI): 41.0-44.0%] of the Malaysians aged > 18 years were reported to suffer from MetS in a nationwide study 4, and they were at risk of MetS-associated morbidity. The same study also demonstrated that JIS criteria were more sensitive in detecting Malaysians with MetS compared to other definitions 4. Malaysians with MetS defined by JIS criteria were shown to have increased high-sensitivity C-reactive protein level and carotid femoral pulse wave velocity (reflective of aortic stiffness), which reflect a higher risk for cardiovascular diseases 5.

Apart from energy metabolism and cardiovascular system, MetS has been implicated in the development of osteoporosis, which is a metabolic bone disease characterized by deterioration of skeletal mass and microarchitecture 6. Each component of MetS affects bone metabolism distinctly. For example, obesity increases the mechanical load of the body and stimulates bone accrual. Adipose tissue also synthesizes oestrogens and adipokines, which alter the risk of hormone-sensitive diseases 7. A previous study showed that oestrogen mediated the relationship between body mass index and breast cancer 8. However, adipose tissue is also a source of pro-inflammatory cytokines which encourage bone resorption. Diabetes and obesity are associated with bone marrow adipogenesis which deprives mesenchymal stem cells available for osteoblast formation 7. Hypertension increases excretion of calcium, elevating parathyroid secretion and bone resorption 9. Oxidized low-density lipoprotein cholesterol particles and excess free fatty acids in dyslipidaemia can uncouple bone remodelling 10-13, favouring bone resorption.

Considering the complicated relationship between MetS components and bone, it is interesting to understand the overall relationship between MetS and bone health. Several studies in the United States and European countries revealed a positive relationship between MetS and bone mineral density (BMD) assessed by dual-energy X-ray absorptiometry (DXA) 14-16. On the other hand, studies among Korean populations demonstrated a negative relationship between BMD and MetS 17-19. Two meta-analyses conducted on this topic also showed distinct results 20, 21. Xue et al. 20 reported that MetS was related to increased lumbar spine BMD (unadjusted for BMI), especially among the Caucasians. Zhou et al. 21 found that lumbar spine and femoral neck BMD (adjusted for BMI) were higher among subjects without MetS, especially in men. These studies demonstrated that ethnicity, sex and adjustment for BMI influence the relationship between MetS and bone health. Notwithstanding the reports from Korea, Mainland China and Taiwan, data from other parts of Asian are very limited. In Malaysia, a cross-sectional study conducted among Malaysian men using calcaneal quantitative ultrasound (QUS) found no significant relationship between MetS and the calcaneal bone index 22. Since QUS is not the standard in measuring bone mass, this finding warrants validation using DXA.

Therefore, the current study aims to determine the relationship between MetS and BMD measured by DXA in a Malaysian population. This study will help to determine whether MetS is a significant risk factor for osteoporosis among the Malaysian population. Based on the previous observations that obesity is predominantly a positive predictor of bone health among Malaysians 23, 24, we hypothesize that MetS has a protective role in bone health.

## Materials and methods

This cross-sectional study was a part of the more comprehensive bone health study of Malaysian populations conducted from April 2018 to April 2019 25-29. Participants were residents of Klang Valley, Malaysia, aged 40 years or above, recruited through purposive sampling method. The recruitment with specific inclusion and exclusion criteria of the subjects was advertised via local vernacular newspapers, radio broadcasts, flyers and community centre. The exclusion criteria were: (1) subjects diagnosed and treated for bone metabolic diseases, such as osteoporosis, osteomalacia, Paget disease and rickets; (2) subjects with medical conditions affecting their bone health, such as hyperparathyroidism and hypercalcaemia; (3) subjects taking medications which could affect their bone health, such as hormone replacement/ablation therapy, glucocorticoids, thyroid supplements, anticonvulsants, warfarin, thiazide diuretics, chemotherapeutic agents; (4) subjects with mobility issues and need a walking aid; and (5) subjects with metal implants which could interfere with DXA scanning. Written informed consent was obtained from the subjects prior to their participation. The study protocol was reviewed and approved by Universiti Kebangsaan Malaysia Research Ethics Committee (Code: UKM PPI/111/8/JEP 2017-721). The study was performed in accordance with the Declaration of Helsinki (2013).

Subjects completed a questionnaire on their demography details, lifestyle, medical and medication history. Their age, sex and ethnicity were confirmed by information on the identification card. Their physical activity status was assessed using the International Physical Activity Questionnaire (IPAQ), previously validated in Malaysian population 30. They were categorized according to the metabolic equivalents of their physical activity into sedentary, minimally active and health-enhancing physically active (HEPA). For smoking status, current smokers were defined as persons who had smoked five packs of cigarettes in his lifetime and continued to smoke regularly at the time of the study. Former smokers were those who had ceased smoking for more than one month. For alcohol consumption, regular drinkers were defined as persons who consumed at least one unit of alcoholic beverages once a week. Former drinkers were defined as persons who had consumed alcohol beverages regularly but had stopped for one month. For dairy consumption, regular dairy users were defined as persons who consumed one unit of dairy products [milk (1 unit = 200 mL), cheese (1 unit = 1 slide) and yoghurts (1 unit = 1 cup)] at least three times a week. In the analysis, the current and former users of cigarettes and alcoholic beverages were combined as 'ever users' due to the small number of formers users.

The standing height of the subjects without shoes was measured using a stadiometer and recorded to the nearest 1 cm (Seca, Hamburg, Germany). The body weight of the subjects with light clothing was measured using a weight scale (Tanita, Tokyo, Japan) and recorded to the nearest 0.1 kg. The body mass index (BMI) was calculated by dividing the body weight in kg by squared height in m. Waist circumference was measured at the midpoint between the lowest rib and the iliac crest using a measuring tape. Blood pressure was measured using an electronic sphygmomanometer (HEM-7120, Omron, Kyoto, Japan) in a sitting position. If blood pressure was elevated (systolic > 130 mmHg and/or diastolic > 85 mmHg), the subjects were rested in a sitting position for 15 mins before a second measurement was performed.

Bone mineral density (BMD) of the subjects at the hip and spine was assessed using DXA (Hologic Discovery QDRWi densitometer, Hologic, MA, USA). Daily calibration was performed using a phantom. A trained technician blinded to the metabolic profile of the subjects positioned the subjects and performed all DXA scans. The short-term *in vivo* coefficient of variation for the DXA machine was 1.8% and 1.2% for lumbar spine and total hip 31. The subjects were categorized into osteoporosis based on a T-score ≤ -2.5 of either lumbar spine or total hip.

Subjects were required to fast for at least eight hours before blood collection, which was conducted between 8 and 10 am. Five millilitres of venous blood was collected by an experienced phlebotomist using heparin-coated tubes. The blood samples were sent immediately to an external accredited laboratory for analysis. The fasting blood glucose, triglyceride, high-density lipoprotein cholesterol and total cholesterol levels were measured based on enzymatic and colourimetric methods using a Cobas^®^ 702 auto-analyzer (Roche, Basel, Switzerland).

### Definition of MetS

MetS was defined using criteria from JIS 3, whereby subjects fulfilling at least three of the following criteria were classified as having MetS: (1) a waist circumference ≥ 90 for men, ≥ 80 cm for women; (2) a systolic blood pressure ≥ 130 mmHg and/or a diastolic pressure ≥ 85 mmHg, or the use of any anti-hypertensive agents; (3); a fasting blood glucose ≥ 5.5 mmol/L, or the use of any anti-diabetic agents; (4); a triglyceride level ≥ 1.7 mmol/L, or the use of any triglyceride-lowering agents (5); an HDL-c level < 1.0 mmol/L in men, <1.3 mmol/L in women or the use of any agents to improve HDL-c level.

### Statistical analysis

Normality of the data was determined using the Kolmogorov-Smirnov test. Comparison of mean BMD based on MetS status of the subjects was performed using univariate analysis. The data were presented as adjusted mean and standard deviation. Multiple linear regression was used to analyze the association between BMD and MetS or its components. The data were presented as standardized regression coefficient (95% CI). Binary logistic regression was used to analyze the association between the occurrence of osteoporosis and MetS or its component. The data were presented as odds ratio (95% CI). Multivariate outliers marked by standardized residual values > 3 were removed in regression models. All analysis was adjusted to the following confounders: (i) Model 1: age, sex, ethnicity; (2) Model 2: confounders in Model 1 and physical activity status, smoking status, alcohol consumption and dairy consumption status; (3) Model 3: confounders in Model 2 and BMI. A p-value < 0.05 was considered as statistical significance. The analysis was performed using SPSS version 25 (IBM, Armonk, USA).

## Results

A total of 190 men [mean age: 57.8 (9.6) years] and 210 women [mean age: 56.1 (8.1) years] participated in the study. Majority of the subjects are Chinese, followed by Malays and Indians/others. MetS was found in 30% [95% CI: 25.6-34.8%] of the subjects, whereas 12.3% (95% CI: 9.2-15.9%) of the subjects were found to have osteoporosis (**Table 1**).

The subjects were ranked according to the number of MetS components (**Table 2**). Subjects with four MetS components showed the highest spine (*p=*0.002) and total hip BMD (*p<*0.001) compared to subjects with zero or one component after adjustment for age, sex and ethnicity. The difference persisted with further adjustment for lifestyle factors, i.e. physical activity status, smoking status, alcohol intake and dairy intake (*p=*0.003 for spine; *p<*0.001 for hip), but was attenuated with further adjustment for BMI (*p=*0.059 for spine; *p=*0.060 for hip). Similarly, spine and hip BMD was higher among subjects with MetS compared to those without after adjustment for potential confounders (*p=*0.006 for spine; *p<*0.001 for hip). However, the difference was attenuated after further adjustment for BMI (*p=*0.625 for spine; *p=*0.478 for hip).

The relationships between MetS or its components and BMD were analyzed using multiple linear regressions (**Table 3**). Waist circumference was associated positively with spine and hip BMD in models adjusted for confounders in Model 1 (*p=*0.009 for spine; *p<*0.001 for hip) and 2 (*p=*0.012 for spine; *p<*0.001 for hip). With further adjustment for BMI, the association between BMD and waist circumference remained significant but switched to negative (*p=*0.044 for spine; *p=*0.021 for hip). Systolic blood pressure was positively associated with total hip BMD (*p=*0.014) but the relationship was attenuated with adjustment for BMI (*p=*0.080). Other components of MetS were not significantly associated with spine and hip BMD (*p*>0.05).

Logistic regression was used to examine the relationship between the presence of osteoporosis and MetS or its components (**Table 4**). A significant negative association between osteoporosis and waist circumference was observed with adjustment for confounders in Model 1 (*p=*0.040) and 2 (*p=*0.002). However, the association was attenuated with further adjustment for BMI (*p=*0.114). Triglyceride level was associated positively with MetS in a fully adjusted model (*p=*0.001). In another logistic regression model, MetS was significantly associated negatively with osteoporosis after adjustment confounders in Model 1 (*p=*0.019), but further adjustment for lifestyle factors and BMI attenuated the relationship (*p*>0.05).

## Discussion

The current study revealed that MetS was associated with higher BMD in the Malaysian population, and this relationship was mediated by BMI. Waist circumference was the MetS component most prominently associated with increased BMD, but adjustment for BMI reversed the association. A higher systolic blood pressure was explicitly associated with increased total hip BMD, and the adjustment for BMI attenuated the relationship. Triglyceride level was associated positively with osteoporosis. Overall, MetS and waist circumference associated negatively with osteoporosis in a model adjusted for age, sex and ethnicity.

The prevalence of MetS in the current study was 30% (95% CI: 25.6-34.8%), which is similar to the national prevalence value reported by Rampal et al. 32 [27.5% (95% CI: 26.8-28.2%), JIS criteria] but lower compared to the Malaysian national prevalence value [42.5% (95% CI: 41.0-44.0%), JIS criteria] and urban prevalence value [44.5% (95% CI: 42.8-46.9%), JIS criteria] previously reported by Mohamud et al. 4. On the other hand, there are limited studies on the prevalence of osteoporosis in Malaysia apart from data reported by our group 26. A study by Lim et al. 33 showed that 24.1% (95% CI: 20.5-28.1%) of the Malaysian women > 45 years (n=514) sampled in Klang Valley had osteoporosis, which is higher compared to 18.1% (95% CI: 13.1-24.0%) found in the current study. Selection bias cannot be excluded in this study, whereby participants who volunteered for health screening could be more health-conscious, therefore had a lower prevalence of MetS and osteoporosis.

The positive relationship between MetS and BMD observed in this study agrees with several previous studies. In the Third National Health and Nutrition Examination Survey III, a positive relationship between MetS and femoral neck BMD was observed in 8197 Americans > 20 years 15. The Camargo Cohort Study (1508 subjects > 50 years) further demonstrated that the positive association between MetS and BMD was observed only in Spanish women but not in men 14. The Berlin Aging Study II [1402 subjects aged 68 (SD 4) years] concurred this observation 34. Sub-analysis based on sex was not performed in this study due to the limited sample size, so sex differences in the relationship between MetS and BMD cannot be confirmed in our subjects. Adjustment for BMI attenuated the relationship between MetS and BMD in this study, which confirms the findings in previous studies 14, 15. This observation suggests that the relationship of MetS on BMD among our subjects is primarily mediated by BMI. Increased BMI could reflect higher mechanical loading on the bone, which stimulate accrual of bone mass. However, other researchers contested BMI-adjustment because obesity is a central component of MetS; therefore, BMI-adjustment fundamentally alters the relationship between MetS and BMD 20. Other studies demonstrated a negative relationship between MetS and BMD 19, 21. In the Korea National Health and Nutrition Examination Survey (n=14485), BMD of men with MetS was lower compared to those without MetS, but no difference was observed in women 19. In a meta-analysis by Zhou et al. 21 compiling nine studies, lumbar spine and femoral neck BMD were higher in non-MetS subjects. They also suggest that MetS exerted an adverse effect on BMD in men but not women 21. Other researchers proposed that in non-Caucasian populations with higher adiposity at a given BMI, such as East Asians, the relationship between MetS and BMD is dominated by the adverse effects of adiposity on bone rather than mechanical loading 35.

The associations between individual components of MetS and BMD were also examined in this study. Waist circumference was associated with BMD positively, but BMI-adjustment changed the direction of association to negative. We suggest that before BMI adjustment, waist circumference is a surrogate of BMI, which reflects the effects of mechanical loading on the skeletal system. After BMI-adjustment, waist circumference is reflective of the degree of adiposity of the body, which has a negative effect on the bone, thus explaining the switching of the direction of the association. The negative association of waist circumference or body fat on bone health indicated by BMD or quantitative ultrasound have been demonstrated by multiple cross-sectional studies previously 19, 23, 24, 36. A similar explanation could be applied to our observation that waist circumference was negatively associated with osteoporosis in the logistic models adjusted for BMI. After BMI-adjustment, the association was attenuated. Kim and Kim 37 showed that osteoporosis risk was lower in obesity defined by BMI (> 25 kg/m^2^) alone or in combination with waist circumference (>80 cm) but higher in obesity defined by waist circumference alone in Korean postmenopausal women (n=3058). Similarly, Zhao et al. reported that fat mass was negatively correlated with bone mass environmentally and genetically, after controlling the effects of body weight, among Chinese and Caucasian subjects 38. In contrast, a higher waist circumference predicted less bone loss in Korean postmenopausal women (n=1218) in a three-year retrospective study, after adjusted for weight and height 39.

Systolic blood pressure was associated positively with total hip BMD in models unadjusted for BMI but not with spine BMD. It was also not associated with osteoporosis in logistics models. Diastolic blood pressure was not associated with BMD or osteoporosis. This observation contradicts the findings of a meta-analysis, which showed that blood pressure is negatively associated with BMD and positively with increased risk of osteoporosis 40. It is generally agreed that high blood pressure increases the excretion of calcium, thus elevating parathyroid hormone level and bone resorption 9. We suggest that increased systolic blood pressure is reflective of increased body size, which increases the peripheral resistance of the vascular system 41, thus explaining the positive association with BMD.

Triglyceride level was associated with an increased risk of osteoporosis in the fully adjusted model, despite a large confidence interval. Dyslipidaemia can be a result of high fat and high carbohydrate intake. Excess free fatty acids could promote osteoclast survival 12 and suppress osteoblast apoptosis 42. Dyslipidaemia may also indicate dysregulation of mevalonate pathway, which is involved in cholesterol synthesis. Isoprenoids are products of the mevalonate pathway used in the synthesis of prenylated proteins, such as Rac, Ras and RhoA, which in turn inhibit bone morphogenetic protein signalling and osteoblastogenesis 43. The study by Kim et al. (1108 postmenopausal women) and Huang et al. (n=2548, aged > 18 years) on Korean women showed that high triglyceride level was associated with lower BMD 44, 45. In a multivariate model, a high triglyceride level was associated with a higher rate of bone loss in Korean postmenopausal women 39. Nevertheless, another study showed that a high triglyceride level was related to a low BMD in men but a high BMD in postmenopausal women in Korea 18. The authors did not explain the sex-specific trend of this relationship, but it could be mediated by sex hormones, which regulate lipid and bone metabolism. The negative relationship between BMD and triglycerides in men could be confounded by variation of sex hormone level in men who still retain their gonadal function till old age. The positive relationship in women could be confounded by body size, in which the excess triglycerides were stored as adipose tissue, thereby increasing body weight. Besides, triglycerides are transported by circulating apolipoprotein E (ApoE), especially ApoE4 46, which `have been implicated in regulating bone metabolism 47. ApoE knockout mice given normal diet were reported to have higher bone mass due to increased bone formation 48, but their bone mass deteriorated faster than the wildtype when challenged with high fat diet 49. However, ApoE expression of the subjects was not determined in this study, thus the role of ApoE in mediating the relationship between triglycerides and BMD remains speculative.

Other components of MetS, such as fasting blood glucose and HDL-c levels, were not associated with BMD or osteoporosis in this study. Nevertheless, other studies have shown that variation in fasting blood glucose is associated with increased BMD 18, 36, 50. Insulin is a bone anabolic hormone due to its homology with insulin-like growth factor-1, and increased insulin secretion during insulin resistance can drive bone formation 51. Advanced glycation products in the bone also impede osteoblast differentiation and function 52, while their effects on osteoclasts depend on the stage of maturation 53. Non-enzymatic glycation of collagen fibres may also alter the mechanical strength of the bone and increase the fracture risk of patients with diabetes 54. HDL-c may ameliorate oxidized LDL-c, which can induce apoptosis of osteoblast and increase osteoclast survival, thus protecting bone health 55. Jeon et al. 17 showed that HDL-c was positively associated with lumbar spine BMD in postmenopausal Korean women (n=931, > 45 years). A similar observation was obtained in the women (n=2040, aged 72.38±6.81 years) but not men [n=1510, aged 72.04 (SD 6.51) years] of The Rotterdam Study 36. In a separate study, low HDL-c was associated with low total hip and lumbar spine BMD in pre-menopausal Korean women [n=4575, aged 35.2 (standard error 0.2) years] 35. However, a Korean study reporting an inverse association between HDL-c and BMD also exist 56.

Overall, the current study showed that MetS was negatively associated with osteoporosis in a logistic model adjusted for age, sex and ethnicity. However, the association did not persist with subsequent adjustment for lifestyle factors and BMI. Therefore, it might not be a strong risk factor for osteoporosis in the Malaysian population. A retrospective study among Taiwanese population [n=2007, aged > 50 years] also reported no association between MetS and osteoporosis in both sexes 57. In contrast, Chen et al. 58 showed that both MetS and non-alcoholic fatty liver disease associated with it were positively associated with osteoporosis among postmenopausal women from Eastern China [n=938, aged 61.2 (SD 13.8) years].

Several limitations prevent the generalization of the results of this study. The small sample size might attenuate the strength of the association between bone health and metabolic outcomes. Sub-analysis based on sex was not performed due to the limited sample size. Menopause status was not included as a confounding variable because it was not a shared characteristic for men and women. The glycaemic status of the subjects was evaluated by fasting blood glucose only, but not confirmed by insulin level and homeostatic model assessment of insulin resistance. The factors mediating the relationship between MetS and BMD, such as pro-inflammatory cytokines, adipokines and sex hormones, were not evaluated in this study. We did not exclude subjects with dietary supplements because the subjects did not show consistent intake. We also did not perform a comprehensive dietary intake survey of the subjects, so the models were not adjusted for energy or nutrients intake. Apart from that, additional assessment of circulating bone formation and resorption markers could help to improve the understanding of MetS on bone metabolism. The current study only examines the association between bone mass and metabolic parameters. Other determinants of bone strength, such as bone geometry and structure, could be examined in the future using peripheral quantitative computed tomography. Due to the cross-sectional nature of the study, the causal relationship between metabolic syndrome and bone health cannot be determined. This could be addressed by a more comprehensive longitudinal study in the future.

## Conclusion

MetS is associated with increased BMD in the Malaysian population, probably due to increased mechanical loading on the bone. Despite that, central adiposity, marked by increased waist circumference, is negatively associated with BMD. Therefore, while MetS may not represent a significant risk of osteoporosis, obesity management may improve metabolic and skeletal health of middle-aged and elderly Malaysians.

## Figures and Tables

**Table 1:** Basic characteristics of the subjects

Characteristics	Male (n=190)	Female (n=210)	Overall (n=400)
	mean (SD)	mean (SD)	mean (SD)
Age (years)	57.8 (9.6)	56.1 (8.1)	56.9 (8.9)
Waist circumference (cm)	88.6 (12.4)	82.2 (10.5)	85.2 (11.9)
Height (cm)	167.1 (6.0)	154.5 (5.4)	160.5 (8.5)
Weight (kg)	70.8 (11.6)	60.1 (11.9)	65.2 (12.9)
Body mass index (kg/m^2^)	25.3 (3.9)	25.2 (5.0)	25.3 (4.5)
Spine BMD (g/cm^2^)	1.00 (0.16)	0.90 (0.16)	0.95 (0.17)
Total hip BMD (g/cm^2^)	0.93 (0.13)	0.83 (0.12)	0.88 (0.14)
Fat mass (kg)	21.0 (6.2)	24.2 (7.3)	22.7 (7.0)
Lean mass (kg)	47.0 (6.2)	33.6 (5.1)	40.0 (8.8)
Fat percentage (%)	29.6 (4.9)	40.1 (5.4)	35.1 (7.4)
Systolic blood pressure (mmHg)	131.9 (18.0)	126.6 (18.2)	129.1 (18.3)
Diastolic blood pressure (mmHg)	85.3 (9.7)	80.7 (10.7)	82.9 (10.5)
Fasting blood glucose (mmol/L)	5.4 (1.5)	5.2 (1.4)	5.3 (1.5)
Serum triglyceride (mmol/L)	1.6 (0.8)	1.3 (0.7)	1.4 (0.8)
Serum HDL-c (mmol/L)	1.3 (0.3)	1.6 (0.4)	1.5 (0.4)
Serum LDL-c (mmol/L)	3.2 (1.1)	3.2 (1.0)	3.2 (1.0)
Serum total cholesterol (mmol/L)	5.2 (1.1)	5.5 (1.0)	5.4 (1.0)
HDL-c/LDL-c ratio	4.2 (1.1)	3.6 (1.1)	3.9 (1.2)
	**n (%)**	**n (%)**	**n (%)**
**Ethnicity**			
Malay	79 (41.6)	90 (42.9)	169 (42.3)
Chinese	91 (47.9)	102 (48.6)	193 (48.3)
Indian or others	20 (10.5)	18 (8.6)	38 (9.5)
**Body mass index**			
Underweight	16 (8.4)	24 (11.4)	40 (10.0)
Normal	88 (46.3)	95 (45.2)	183 (45.8)
Overweight	86 (45.3)	91 (43.3)	177 (44.3)
**Physical activity**			
Sedentary	80 (42.1)	99 (47.1)	179 (44.8)
Minimally active	73 (38.4)	85 (40.5)	158 (39.5)
HEPA-active	37 (19.5)	26 (12.4)	63 (15.8)
**Cigarette smoker**			
Yes	78 (41.1)	7 (3.3)	85 (21.3)
No	112 (58.9)	203 (96.7)	315 (78.8)
**Ever an alcoholic drinker**			
Yes	65 (34.2)	37 (17.6)	102 (25.5)
No	125 (65.8)	173 (82.4)	298 (74.5)
**Regular daily consumers**			
Yes	137 (72.1)	113 (53.8)	150 (37.5)
No	53 (27.9)	97 (46.2)	250 (62.5)
**Osteoporosis**			
Osteoporosis (T-score ≤ -2.5)	11 (5.8)	38 (18.1)	49 (12.3)
Osteopenia (T-score between -1 and -2.4)	68 (35.8)	101 (48.1)	169 (42.3)
Normal (T-score > -1)	111 (58.4)	71 (33.8)	182 (45.5)
**Metabolic syndrome**			
Yes	59 (31.1)	149 (71.0)	120 (30.0)
No	131 (68.9)	61 (29.0)	280 (70.0)
**Increased waist circumference^1^**			
Yes	85 (44.7)	127 (60.5)	212 (53.0)
No	105 (55.3)	83 (39.5)	188 (47.0)
**Increased blood pressure^2^**			
Yes	132 (69.5)	109 (51.9)	241 (60.2)
No	58 (30.5)	101 (48.1)	159 (39.8)
**Increased fasting blood glucose^3^**			
Yes	56 (29.5)	44 (21.0)	100 (25.0)
No	134 (70.5)	166 (79.0)	300 (75.0)
**Increased triglyceride level^4^**			
Yes	71 (37.4)	56 (26.7)	127 (31.8)
No	119 (62.6)	154 (73.3)	273 (68.3)
**Reduced HDL-c^5^**			
Yes	22 (11.6)	44 (21.0)	66 (16.5)
No	168 (88.4)	166 (79.0)	334 (83.5)

Notes: (1) increased waist circumference: ≥ 90 for men, ≥ 80 cm for women; (2) increased blood pressure: systolic blood pressure ≥ 130 mmHg and/or a diastolic pressure ≥ 85 mmHg, or the use of any anti-hypertensive agents; (3); increased fasting blood glucose: ≥ 5.5 mmol/L, or the use of any anti-diabetic agents; (4); increased: triglyceride level ≥ 1.7 mmol/L, or the use of any triglyceride-lowering agents (5); reduced HDL-c level: < 1.0 mmol/L in men, <1.3 mmol/L in women or the use of any agents to improve HDL-c level.

**Table 2:** Bone mineral density of the subjects according to the status of metabolic syndrome

No. of MetS component	Adjusted mean (SD) of spine BMD			Adjusted mean (SD) of hip BMD		
	Model 1	Model 2	Model 3	Model 1	Model 2	Model 3
0 (n=69)	0.93 (0.16)	0.93 (0.17)	0.97 (0.17)	0.85 (0.12)	0.84 (0.12)	0.89 (0.12)
1 (n=99)	0.91 (0.15)	0.91 (0.16)	0.93 (0.16)	0.84 (0.12)	0.84 (0.12)	0.86 (0.11)
2 (n=112)	0.96 (0.16)	0.95 (0.16)	0.94 (0.15)	0.89 (0.12)^b^	0.89 (0.12)	0.88 (0.11)
3 (n=68)	0.95 (0.16)	0.95 (0.16)	0.92 (0.16)	0.89 (0.12)	0.89 (0.12)	0.86 (0.12)
4 (n=41)	1.03 (0.15)^ab^	1.03 (0.15)^ab^	1.00 (0.15)	0.96 (0.12)^abc^	0.96 (0.12)^abc^	0.92 (0.12)
5 (n=11)	1.03 (0.15)	1.00 (0.16)	0.98 (0.15)	0.88 (0.12)	0.88 (0.12)	0.86 (0.11)
*p*	**0.002**	**0.003**	0.059	**<0.001**	**<0.001**	0.060
**Presence of MetS**						
No (n=280)	0.94 (0.15)	0.93 (0.15)	0.95 (0.15)	0.86 (0.12)	0.86 (0.12)	0.87 (0.12)
Yes (n=120)	0.98 (0.15)	0.98 (0.15)	0.96 (0.16)	0.91 (0.12)	0.91 (0.12)	0.88 (0.12)
*p*	**0.008**	**0.006**	0.625	**<0.001**	**<0.001**	0.478

The letter 'a' indicates a significant difference (*p<*0.05) compared to '0 MetS component'; 'b' compared to '1 MetS component'; 'c' compared to '2 MetS components'. Model 1: adjusted for age, sex, ethnicity; Model 2: adjusted for Model 1 + physical activity status, cigarette-smoking status, alcohol-drinking status, dairy-consumption status; Model 3: adjusted for Model 2 + body mass index. Abbreviation: BMD, bone mineral density; MetS, metabolic syndrome; n, sample size; SD, standard deviation.

**Table 3:** The relationship between metabolic syndrome/its components and bone mineral density

	Spine BMD									Hip BMD								
	Model 1			Model 2			Model 3			Model 1			Model 2			Model 3		
	β	95 % CI	*p*	β	95 % CI	*p*	β	95 % CI	*p*	β	95 % CI	*p*	β	95 % CI	*p*	β	95 % CI	*p*
Waist circumference	0.153	L: 0.039 U: 0.266	**0.009**	0.149	L: 0.003 U: 0.295	**0.012**	-0.183	L: -0.361 U: -0.005	**0.044**	0.234	L: 0.134 U: 0.333	**<0.001**	0.219	L: 0.076 U: 0.362	**<0.001**	-0.179	L: -0.330 U: -0.028	**0.021**
Systolic blood pressure	0.07	L: -0.080 U: 0.221	0.359	0.095	L: -0.091 U: 0.281	0.223	0.043	L: -0.107 U: 0.193	0.577	0.158	L: 0.026 U: 0.290	**0.019**	0.168	L: -0.161 U: 0.497	**0.014**	0.114	L: -0.013 U: 0.242	0.080
Diastolic blood pressure	0.048	L: -0.099 U: 0.195	0.525	0.029	L: -0.010 U: 0.156	0.702	0.018	L: -0.127 U: 0.163	0.808	-0.092	L: -0.222 U: 0.038	0.165	-0.099	L: -0.293 U: 0.095	0.134	-0.092	L: -0.215 U: 0.032	0.147
Fasting blood glucose	0.039	L: -0.060 U: 0.139	0.437	0.011	L: -0.075 U: 0.097	0.833	0.006	L: -0.093 U: 0.105	0.904	0.062	L: -0.025 U: 0.149	0.166	0.037	L: -0.050 U: 0.124	0.413	0.013	L: -0.071 U: 0.097	0.759
Serum triglyceride	-0.024	L: -0.127 U: 0.079	0.647	-0.001	L: -0.174 U: 0.172	0.991	-0.015	L: -0.118 U: 0.088	0.772	0.035	L: -0.056 U: 0.125	0.456	0.042	L: -0.040 U: 0.124	0.366	0.04	L: -0.048 U: 0.127	0.377
Serum HDL-c	-0.009	L: -0.128 U: 0.110	0.877	0.001	L: -0.050 U: 0.052	0.981	-0.007	L: -0.127 U: 0.113	0.905	-0.038	L: -0.143 U: 0.066	0.473	-0.048	L: -0.153 U: 0.057	0.378	-0.035	L: -0.137 U: 0.067	0.497
n*	**387**			**384**			**382**			**388**			**385**			**382**		
MetS	0.127	L: 0.035 U: 0.219	**0.008**	0.128	L: 0.035 U: 0.0221	**0.008**	0.009	L: -0.097 U: 0.115	0.851	0.163	L: 0.078 U: 0.246	**<0.001**	0.165	L: 0.081 U: 0.249	**<0.001**	0.006	L: -0.079 U: 0.091	0.896
n*	**400**			**400**			**396**			**400**			**400**			**395**		

*the sample size is < 400 because multivariate outliers were removed. L: Lower 95% CI; U: Upper 95% CI. Model 1: adjusted for age, sex, ethnicity; Model 2: adjusted for Model 1 + physical activity status, cigarette-smoking status, alcohol-drinking status, dairy-consumption status; Model 3: adjusted for Model 2 + body mass index. Abbreviation: β, standardized regression coefficient; BMD, bone mineral density; HDL-c, high-density lipoprotein cholesterol; MetS, metabolic syndrome; n, sample size.

**Table 4:** The relationship between metabolic syndrome/its components and osteoporosis

	Model 1			Model 2			Model 3		
	OR	95% CI	*p*	OR	95% CI	*p*	OR	95% CI	*p*
Waist circumference	0.962	0.927-0.998	**0.040**	0.915	0.865-0.968	**0.002**	1.094	0.979-1.224	0.114
Systolic blood pressure	0.971	0.937-1.007	0.111	0.971	0.931-1.012	0.163	1.013	0.963-1.066	0.614
Diastolic blood pressure	1.032	0.972-1.097	0.304	1.020	0.950-1.094	0.589	0.938	0.842-1.044	0.242
Fasting blood glucose	0.947	0.716-1.252	0.701	0.853	0.584-1.245	0.409	1.066	0.618-1.838	0.819
Serum triglyceride	1.523	0.928-2.499	0.096	1.860	1.095-3.160	0.022	4.693	1.842-11.956	**0.001**
Serum HDL-c	1.14	0.414-3.139	0.801	1.495	0.506-4.416	0.467	0.950	0.234-3.858	0.943
n*	396			386			382		
MetS	0.209	0.057-0.770	**0.019**	0.545	0.221-1.346	0.188	0.907	0.186-4.411	0.904
n*	380			391			383		

*the sample size is < 400 because multivariate outliers were removed. Model 1: adjusted for age, sex, ethnicity; Model 2: adjusted for Model 1 + physical activity status, cigarette-smoking status, alcohol-drinking status, dairy-consumption status; Model 3: adjusted for Model 2 + body mass index. Abbreviation: CI, confidence interval; HDL-c, high-density lipoprotein cholesterol; MetS, metabolic syndrome; n, sample size; OR, odds ratio.
